# Determinants of Intention to Use HIV Pre-exposure Prophylaxis and Condom Use Among a Sample of Cisgender Female Sex Workers Working Mostly Outdoors in Madrid, Spain

**DOI:** 10.1007/s10508-024-02834-4

**Published:** 2024-06-10

**Authors:** Laia J. Vazquez Guillamet, Jorge Valencia, Pablo Ryan, Guillermo Cuevas-Tascón, Miguel Angel del-Olmo-Morales, Ines Cobo, Jeffrey V. Lazarus, Guillaume Chevance

**Affiliations:** 1 Health Systems Research Group, Barcelona Institute for Global Health (ISGlobal), Hospital Clínic, University of Barcelona, Calle del Rossellón 171, 1 Floor, ES 08036 Barcelona, Spain; 2 eHealth Group, Barcelona Institute for Global Health (ISGlobal), Hospital Clínic, University of Barcelona, Barcelona, Spain; 3 Fundación de Investigación Biomédica, Hospital Infanta Leonor, Madrid, Spain; 4 Madrid Positivo Non-Governmental Organization, Madrid, Spain; 5 Departamento de Medicina Interna, Hospital Infanta Leonor, Madrid, Spain; 6 Centro de Investigación Biomédica en Red en Enfermedades Infecciosas, Madrid, Spain; 7 Asociación in Genero (Interculturalidad y Género), Ciudad Real, Spain; 8 Faculty of Medicine, Hospital Clínic, University of Barcelona, Barcelona, Spain; 9 Facultat de Medicina i Ciencies de la Salut, Universitat de Barcelona (UB), c. Casanova 143, 08036 Barcelona, Spain

**Keywords:** HIV, Women's health services, Sex work, Pre-exposure prophylaxis (PrEP), Condoms, Stigma

## Abstract

There is scant knowledge regarding pre-exposure prophylaxis (PrEP) use among female sex workers (FSWs) in Europe. Spain recognized FSWs as a population at high risk of acquiring HIV and granted them subsidized access to PrEP when the medication first became nationally available in 2019. Nevertheless, FSWs represented just 0.2% of PrEP users in 2022. A total of 102 HIV-negative FSWs reached through field activities of local NGOs located in Madrid were interviewed between January and March 2022. Participants were selected through convenience sampling over a fixed recruitment period. FSWs completed a 73-item survey with questions about individual, occupational, social, and structural determinants. The objective of this study was to identify (1) the prevalence of intention to use oral PrEP and its determinants, and (2) the prevalence of inconsistent condom use, which is the risk factor that qualifies FSWs for subsidized PrEP in the national health system, and its determinants. Importantly, the study sample overrepresented street-based FSWs (71.6%). A quarter (25.5%) of the study participants used condoms inconsistently. PrEP awareness was low (9.8%), but intention to use PrEP was high (72.5%). Intention to use oral PrEP was significantly associated with feeling protected against HIV by taking PrEP and perceiving insufficient protection by condom use alone. Inconsistent condom use was significantly associated with frequent heroin/cocaine use, having clients who inject drugs, and willingness to take PrEP despite it not protecting 100% against HIV infection. FSWs, in this specific sample, are likely to benefit from targeted PrEP awareness campaigns and implementation projects that prioritize those who use drugs and are more likely to engage in condomless sex.

## Introduction

Since its initial recommendation by the World Health Organization in 2012, pre-exposure prophylaxis (PrEP) has emerged as an empowering tool for HIV prevention among at-risk populations. One such population is female sex workers (FSWs), who represented an estimated 10.4% of the global HIV burden in 2018 (Shannon et al., 2018). The available evidence shows that PrEP awareness among FSWs is low, but interest is high (Restar et al., 2017; Robertson et al., 2013; Tomko et al., 2019; Vazquez Guillamet et al., 2024; Ye et al., 2014), and adherence is unpredictable (Ghayda et al., 2020). Past studies investigating PrEP usage among FSWs have included participants from sub-Saharan Africa, Asia, Mexico, and the USA (Ghayda et al., 2020; The Foundation for AIDS Research, 2017), limiting the generalization of results to the European and specifically the Spanish context. In addition, few studies have researched FSWs’ preferences between the different PrEP delivery formats: oral, injectable, and vaginal (Mantsios et al., 2022; Peitzmeier et al., 2017). The lack of European PrEP campaigns and research addressing women (Cairns, 2021), and FSWs in particular (Hayes et al., 2019), might be explained by the low prevalence of HIV in this population compared to men who have sex with men (MSM), especially in western Europe (0–2%) (European Centre for Disease Prevention & Control, 2015). However, to achieve the ambitious goal of ending the AIDS pandemic by 2030 (World Health Organization. Regional Office for Europe, 2017), it is necessary to target all the populations at risk of HIV infection.

Spain recognized FSWs as a population at high risk of acquiring HIV and granted them subsidized access to PrEP when the medication first became nationally available in 2019. PrEP is marketed in Spain in the form of daily pills and delivered in hospital-based pharmacies and authorized healthcare centers (Grupo de trabajo de PrEP. División de Control de VIH, ITS, Hepatitis virales y Tuberculosis. Ministerio de Sanidad, 2021). Nevertheless, FSWs represented just 0.2% of PrEP users in Spain in 2022 (División de Control de VIH, ITS, Hepatitis virales y Tuberculosis. Sistema de información de programas de Profilaxis Pre-exposición al VIH en España (SIPrEP), 2022). Today, there are no existing PrEP community programs or mobile units adapted to the needs of sex workers.

There is not a single theory to guide PrEP usage, with many influencing internal and external factors to be taken into account that are particular to FSWs. The Information-Motivation-Behavioral Skills model for PrEP uptake highlights the importance of accurate PrEP knowledge, motivation to act on PrEP knowledge, and acquisition or maintenance of necessary behavioral skills to successfully overcome obstacles to initiate PrEP (Dubov et al., 2018). Additionally, the structural vulnerability theory helps understand why HIV risk and preventive behaviors might be constrained by existing power structures, elevating the risk of negative health outcomes (Tomko et al., 2019). In alignment with these theories, there is a significant body of research highlighting the relationship between individual (i.e., age, income, sexual health, substance abuse, motivation), social (i.e., norms, social cohesion, social support, stigma regarding work, HIV, and/or PrEP), occupational (i.e., location of sex work, number of clients, clients request of substance use, condom use), and structural determinants (i.e., access to social and health services, attitudes from healthcare providers, migrant status, regulatory sex laws) and FSWs’ vulnerability to HIV infection and access to appropriate HIV care and prevention (Ghayda et al., 2020; Lyons et al., 2020; Platt et al., 2013; Shannon et al., 2018; Tokar & Naniche, 2020; Tomko et al., 2019).

The purpose of this study was to expand the limited body of knowledge about PrEP usage among FSWs in Spain while providing insight into the main barriers and facilitators to PrEP usage to guide future interventions. The objective of this study was to identify the prevalence of intention to use PrEP and its determinants among a sample of FSWs in Madrid, Spain, in parallel with the prevalence of condom use and its determinants, being inconsistent condom use the risk factor that qualifies FSWs for subsidized PrEP in the national health system. In addition, participants were asked about their preferences for PrEP delivery options.

## Method

### Participants

The study took place in the autonomous community of Madrid, Spain, among street-based and non-street-based FSWs. Street-based FSWs were interviewed at the Marconi Polygon and La Cañada Real, while non-street-based FSWs were interviewed at apartments, houses, hotels, and nightclubs in different areas of Madrid. The Marconi polygon is an area of industrial warehouses where approximately 400 women work in the sex trade. The Cañada Real is the largest shantytown in Europe, with close to 8,000 inhabitants living in a 14.4-km-long stretch. Section VI of this settlement hosts the main drug dealing area of Madrid. The Marconi polygon and La Cañada Real are frequented by mobile units from the NGOs Madrid Positivo and InGenero, which provide outreach health and social services. Both mobile units facilitated this study’s encounters with FSWs.

It is estimated that Spain has between 80,000 and 100,000 FSWs (Meneses-Falcón & Urío, 2021); however, the true population size of FSWs in Spain and Madrid, is unknown. While sex work is not illegal, it is unlawful to carry out activities such as exploitation and pimping. For this study, sex work was defined as the exchange of sex for money or goods.

Street- and non-street-based FSWs were invited to participate in the study if they were: (1) cisgender; (2) HIV-negative with the most recent test performed in the previous 6 months or of unknown HIV status; (3) fluent in spoken Spanish and with a literacy level enough to understand the informed consent written in Spanish with help of the study staff; and (4) willing and able to give informed consent.

### Procedure

This study took place as part of an ongoing screening campaign for HIV targeting hard-to-reach populations in Madrid. The Information-Motivation-Behavioral Skills model for PrEP uptake (Dubov et al., 2018) and the structural vulnerability theory (Tomko et al., 2019) guided the creation of a cross-sectional 73-item survey (see the survey at: https://osf.io/94et3/?view_only=ab29200774d24a5ba659df8ae91d97a3) on demographics, HIV risk, preferences on PrEP delivery, intention to use PrEP, as well as individual, occupational, social, and structural barriers to intention to use oral PrEP and condom use that was delivered in person to FSWs in Madrid from January to March 2022. In the absence of a single PrEP survey tool for FSWs addressing these domains, the survey was developed by combining survey questions used in prior research studies about the factors associated with HIV risk and PrEP use in FSWs (“Development of the Perceived Risk of HIV Scale” n.d.; Guillamet et al., 2019; “HIV Questionnaire” n.d.; “Profilaxis de prexposición | Información básica | VIH/SIDA | CDC” n.d., 2021; Klein & Washington, 2019; Napper et al., 2012; Peitzmeier et al., 2017; Stein et al., 2014; Tomko et al., 2019). Subsequently, the survey was reviewed by personnel from several NGOs related to sex work to obtain their feedback and piloted in a small group of sex workers from the study area before its rollout. It took on average 20 min to complete the final version of the survey and FSWs received an economic incentive of 10 euros for participating.

Women were offered HIV rapid testing performed by healthcare personnel if they had not been tested in the previous six months. For street-based FSWs, these tests were carried out inside the mobile unit from a drop of capillary blood obtained from a fingerprick. For non-street-based FSWs, these tests were carried out in a private environment selected by the women with a swab from the gingival mucosa (OraQuick® HIV) (OraQuick n.d., 2022). Rapid tests were analyzed in situ and discarded after a single use. Women were asked to wait for the results, which took approximately 20 min.

### Measures

#### Dependent Variables

The main study’s outcome was the intention to use oral PrEP. Oral PrEP was the delivery method chosen as the main outcome because it was the only one available in Spain. After a brief introduction to PrEP, participants were asked: How interested would you be in taking a daily pill to prevent HIV infection? (Tomko et al., 2019), to which participants replied using a 5-category Likert scale: very interested, somewhat interested, neutral, not very interested, not interested at all.

The second outcome variable was the frequency of condom use with clients (which we will refer to as condom use), the risk factor that qualifies FSWs for subsidized PrEP in the Spanish national health system. Condom use was asked as follows: In your relationship with clients over the last three months, how often have you used condoms during anal or vaginal sex encounters? (Tomko et al., 2019), to which participants replied using a 5-category Likert scale: never, almost never, occasionally, almost every day, every day. The decision to not include condomless oral sex was based on it being a widespread practice (Lee et al., 2021) and having a low risk of associated HIV infection (Baggaley et al., 2008).

#### Independent Variables

All the items presented and answer modalities are available in the supplemental material (see study survey and legend at: https://osf.io/94et3/?view_only=ab29200774d24a5ba659df8ae91d97a3) and organized below in four different categories according to the literature on this topic.

##### Individual Determinants

Demographic data included age, years as a sex worker, and highest education level attempted. Participants were asked about their use of recreational drugs in the past three months, route of use, and frequency with a Likert scale (never, rarely, occasionally, almost every day, everyday). In addition, participants were asked about alcohol intake in the last three months and its frequency using the same Likert scale. A PHQ-2 (Kroenke et al., 2003) and a PHQ-9 (Kroenke et al., 2001) scales were used for screening and diagnosis of depression, and a single-item questionnaire was used to screen for post-traumatic stress disorder (PTSD) (Gore et al., 2008).

Participants also reported their perceived risk of HIV infection in the previous three months using a Likert scale (null, low, middle, high, very high), and the number of diagnosed sexually transmitted infections (STIs) in the last year. Participants were asked whether they knew about PrEP and post-exposure prophylaxis (PEP) before the study and whether they had taken either of those medications. After a brief introduction about PrEP and the different delivery formats (oral, vaginal, injected), participants were asked to rate their intention to take each one of them using a Likert scale. Finally, participants replied Yes or No to multiple statements inquiring about their perceptions about the need for PrEP, concerns about PrEP efficacy and side effects, competing priorities (i.e., the need for PrEP overshadowed by other daily needs), and inconveniences related to each PrEP delivery route (i.e., being afraid of needles, performing frequent vaginal hygiene, considering that it is difficult to remember to take a pill every day).

##### Occupational, Work-Related, Determinants

Taking into consideration the three months before the interview, participants were asked about their usual sex workplace, average daily income, average number of clients per day, and multiple frequency questions using Likert scale (never, rarely, occasionally, almost every day, everyday) about drug use or alcohol intake with clients prior or during sex encounters and whether this activity led to condomless sex, condom use, condom rupture, condom coercion (whether the client refused using condom or removed it during sex despite having agreed to it beforehand), violent encounters (verbal or physical), and sex encounters with clients who inject drugs. Participants were also asked if they share their occupation with healthcare providers when it is appropriate to do so.

##### Social and Interpersonal Determinants

Taking into consideration the three months before the interview, participants were asked if they believed that their stable sexual partner had sex with other people (stable sexual partners were defined as those who do not pay or give goods in exchange for sex). The same questions about frequency of condom use, condom rupture, condom coercion, violent encounters, and use of injected drugs used for clients were also applied to the stable sexual partners. Finally, participants replied Yes or No to multiple statements inquiring about dependence on external influence (when the use of PrEP is conditioned by the opinion or action of others), HIV stigma (when taking PrEP would cause people to think they are HIV positive), and PrEP-related stigma.

##### Structural Determinants

Participants were asked to report their status of immigration, employment, and homelessness in the previous three months. They also reported their affiliation with sex work organizations and reception of aid from NGOs and/or community programs. Participants were asked if they had been arrested by police in the previous year, and if because of police presence, they had changed their area of work in the last 12 months, had to rush their negotiations with clients, or avoided carrying condoms to not be identified as sex workers (these last two questions only applied to street-based FSWs). Finally, participants reported attendance to primary care and replied Yes or No to multiple statements about barriers to health care such as work schedule, finance, and anticipated stigma from healthcare personnel.

### Statistical Analysis

We did not compute a priori power analyses for this study. Instead, we recruited participants during a fixed recruitment period of 3 months constrained by NGOs’ commitment to other activities. All survey questions with multiple answers were regrouped and coded into a dichotomous variable given that the number of participants in some categories was too small for adequate effect estimation. The dichotomization process depended on the type of variable. Neutral responses found in some Likert scales were interpreted as negative. For example, when participants were asked about PrEP interest, they were classified as intenders if they replied totally agree and agree, and non-intenders if they replied neutral, disagree, or totally disagree. For most frequency-related Likert scales, the answers were dichotomized based on whether the event of interest had ever happened (rarely, occasionally, almost every day, everyday) or not (never). An exception to this rule was questions related to the frequency of condom use, in which answers were dichotomized based on consistent (everyday) or inconsistent (never, rarely, occasionally, almost everyday) condom use. Another exception was the frequency of drug use, dichotomized based on frequent drug use (everyday or almost everyday) versus infrequent and no drug use (never, rarely, occasionally), as this seemed to differentiate women with addiction to drugs versus those who only use drugs for work purposes. A detailed description of the dichotomization of each independent variable is available in the study legend on the Open Science Framework platform (https://osf.io/94et3/?view_only=ab29200774d24a5ba659df8ae91d97a3).

**Fig. 1:**
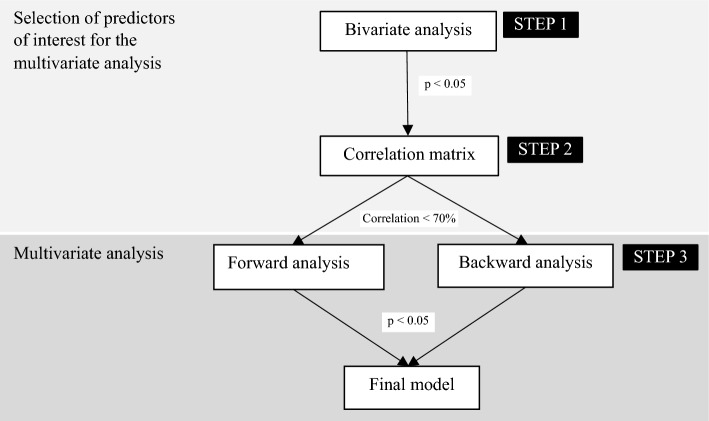
Description of the steps performed in the statistical analysis

Stepwise forward and backward regression analyses were performed to identify variables significantly associated with the outcomes of interest. A selection of independent variables to be included in the final regression models was performed in two steps to reduce the number of candidate variables to be included in the model and avoid issues related to collinearity (see Fig. 1). First, we tested the differences between FSWs who (1) had intention to use PrEP versus no intention to use PrEP and (2) used condoms consistently versus used condoms inconsistently. Chi-square test, Fisher exact test, and Student’s *t* test were used to detect significant differences between groups for all the potential determinants listed in the previous section depending on the variables’ distribution. Second, a correlation matrix including all the variables that significantly differed between the two groups (identified in step 1 previously described) was computed for each outcome variable separately (intention to use PrEP and condom use). When the correlation between two variables was ≥ 70% and the information portrayed by the variables was judged relatively similar, the research team agreed on one of the variables to be kept for the final models to ensure parsimony. Correlation tables are available in the study code https://osf.io/94et3/?view_only=ab29200774d24a5ba659df8ae91d97a3.

Finally, stepwise selections using both forward and backward ordinal regression models were performed (see Fig. 1). The independent variables resulting from the previous two-step selection process were used in the models as candidate variables (“A Note on a General Definition of the Coefficient of Determination on JSTOR”, n.d.), and intention to use PrEP and condom use as outcome variables. Only the determinants that were significantly associated with the two, backward and forward, regression models were discussed as significant correlates of the outcomes. For this final model, the outcome variables intention to use PrEP and condom use were not dichotomized but kept as 5-factor variables. Statistical significance was set at a *p* value < 0.05 for all analyses. Models’ goodness of fit was estimated using a pseudo-R-squared measure designed for multinomial outcomes, the Nagelkerke pseudo-R-squared (Nagelkerke, 1991). Its interpretation is similar to traditional R-squared (i.e., percentage of variance explained by the model relative to the null model). The statistical software R was used for performing the analyses. Following recommended open-science practices and for replication purposes, code and data are available on the Open Science Framework platform (https://osf.io/94et3/?view_only=ab29200774d24a5ba659df8ae91d97a3).

## Results

A total of 102 FSWs completed the questionnaire. Participants had a mean age of 38.7 years (± 10) Most FSWs were migrants (64.7%); 71.6% worked in the street; 45.0% were homeless; 52.0% had used cocaine and/or heroin in the previous three months, 45.0% on a daily or almost daily basis and 20.7% through injection. A fourth of the participants acknowledged inconsistent condom use with clients (25.5%) and condom rupture (26.5%). Half of the participants (52.0%) reported condom coercion by clients during the 3 months prior to the interview.

Five (5.5%, 5/91; 11 missing values for this item) participants knew about post-exposure prophylaxis (PEP), and ten (9.8%) knew about PrEP before the study. Two FSWs (2.9%) had used PEP before, and none had used PrEP. Almost three-quarters of the participants reported being very interested or interested in using PrEP (72.5%), as an oral pill (59.0%), as an injection (57.8%), and as a vaginal ring (22.5%). More information about demographics can be found in Table 1.

**Table 1:** Demographics

	*N* = 102
Age (M ± SD)	38.7 ± 1.1
Migrant	66 (64.7%)
Legal status in Spain	48 (72.7%)
Homelessness in the last 3 months	46 (45.1%)
Education	
Primary school	28 (27.4%)
High school	37 (36.3%)
University studies	11 (10.8%)
Professional training	22 (21.6%)
Years as sex worker (M ± SD)	6.4 ± 0.8
Place of sex work in the last 3 months	
Street	73 (71.6%)
Brothel/Apartment/House	29 (28.4%)
Number of clients per day in the last 3 months (M ± SD)	6 ± 0.5
Monthly income in euros in the last 3 months (M ± SD)	2994 ± 211
Stable sexual partner in the last 3 months	44 (43.1%)
Drug use (heroin and/or cocaine) in the last 3 months	56 (54.94%)
Smoked	46 (45.1%)
Injected	11 (10.8%)
Snored	4 (4%)
Daily or almost daily drug use in the last 3 months	46 (45.0%)
Alcohol use in the last 3 months	30 (29.4%)
Moderate to severe depression (PHQ9)*	36 (35.3%)
Post-traumatic stress disorder (SIPS)**	52 (51.5%)

*Patient Health Questionnaire-9, **Singe-item PTSD screener

### Determinants of Intention to Use PrEP

#### Bivariate Analyses (Step 1)

With regard to individual determinants, there were no significant differences between women who reported intention to use PrEP and women who did not in terms of demographics, substance use, mental health, previously diagnosed STIs, competing priorities, and inconveniences related to PrEP delivery route. Women who expressed intention to use PrEP were significantly more likely than women who reported no intention to use PrEP to report being worried about contracting HIV (90.2% vs. 38.5%), and feeling protected against HIV if taking PrEP (96.7% vs. 76.9%). On the other hand, women who reported intention to use PrEP were less likely to report consistent condom use (18% vs. 63.4%) and concern about side effects (21.3% vs. 43.9%) as reasons for not taking PrEP (Table 2). With regard to occupational, social, or structural determinants, women who reported intention to use PrEP were more likely to be originally from Spain (42.6% vs. 24.4%) (Tables 3, 4, and 5).

**Table 2:** Individual determinants

	Intention to use PrEP			Condom use		
	Intenders (*n* = 61)	Non-intenders (*n* = 41)	*p* value	Consistent (*n* = 77)	Inconsistent (*n* = 25)	*p* value
Demographics						
Age (mean ± standard deviation)	39 ± 11	38.3 ± 10.8	0.7	38.5 ± 11.5	39.6 ± 8.8	0.6
Years as sex worker (mean ± SD)	6.5 ± 6.9	7.1 ± 7.8	0.7	6.3 ± 7.2	8.2 ± 7.4	0.2
High education	18 (29.5%)	14 (34.1%)	0.7	24 (32%)	8 (32%)	1
Substance use						
Drug use (heroin/ cocaine/ marihuana)	33 (54.1%)	23 (56.1%)	0.8	34 (44.2%)	22 (91.7%)	**< 0.001**
Type of drug						
Cocaine	30 (49.2%)	18 (43.9%)	0.6	27 (35.1%)	21 (84%)	**< 0.001**
Heroine	21 (34.4%)	13 (31.7%)	0.8	18 (22.1%)	16 (65%)	**< 0.001**
Daily or almost daily drug use	28 (45.9%)	18 (43.9%)	0.8	24 (31.2%)	22 (91.7%)	**< 0.001**
Route of drug use						
Smoked	26 (42.6%)	20 (48.8%)	0.5	23 (29.8%)	22 (88%)	**< 0.001**
Injected	8 (13.29%)	3 (7.5%)	0.4	5 (6.5%)	6 (24%)	**0.01**
Snored	2 (3.3%)	2 (4.9%)	0.7	4 (5.2%)	0	0.3
Alcohol use	19 (31.2%)	11 (26.8%)	0.6	26 (33.8%)	4 (16%)	0.09
Diagnosis of moderate to severe depression (PHQ9)	25 (41%)	11 (26.8%)	0.1	21 (27.3%)	15 (60%)	**0.003**
Post-traumatic stress disorder (SIPS*)	31 (50.8%)	21 (51.2%)	1	38 (49.4%)	14/24 (58.4%)	0.4
Self-perceived risk of HIV infection in the last 3 months	24 (39.4%)	8 (19.5%)	**0.04**	19 (24.6%)	13 (52%)	**0.01**
Diagnosed with at least one STI in the last year	15 (24.5%)	9 (22%)	0.8	11 (14.3%)	13 (52%)	**< 0.001**
Interest in PrEP and perceptions about PrEP						
Interest in PrEP regardless of PrEP delivery route	61 (100%)	13 (11%)	**< 0.001**	54 (70.1%)	20 (80%)	0.3
Perception of the need for PrEP						
I do not worry about contracting HIV; therefore, I do not need to take PrEP	6 (9.8%)	17 (41.5%)	**< 0.001**	17 (22.1%)	6 (24%)	0.8
I always use condoms; therefore, I do not need PrEP	11 (18%)	26 (63.4%)	**< 0.001**	34 (44.2%)	3 (12%)	**0.004**
Concerns about PrEP efficacy and side effects						
I would feel protected against HIV if I took PrEP	59 (96.7%)	30/39 (76.9%)	**0.003**	67 (87%)	22 (88%)	0.8
I would take PrEP even if it did not protect me 100% against getting infected by HIV	53 (86.8%)	24/40 (60%)	**0.002**	54 (70.2%)	23 (92%)	**0.03**
I would continue using condoms if I took PrEP to protect me against HIV and other STDs	60 (98.3%)	36/39 (92.3%)	0.1	73 (94.8%)	23 (92%)	0.2
I do not want to take PrEP because I worry about side effects	13 (21.3%)	18 (43.9%)	**0.01**	24 (31.2%)	7 (28%)	0.8
Competing priorities						
I am very busy; I do not have time to take PrEP	8 (13.1%)	10 (24.4%)	0.1	14 (18.2%)	4 (16%)	0.8
Inconveniences related to PrEP delivery route						
It is difficult to remember to take pills daily	25 (39%)	24/40 (60%)	**0.06**	38 (49.5%)	11 (44%)	0.6
I do not have a place to keep the pills	14 (23%)	8 (19.5%)	0.7	11 (14.3%)	11 (44%)	**0.002**
I perform regular vaginal hygiene	42 (68.9%)	29 (70.7%)	0.7	58 (75.3%)	13 (52%)	**0.01**
I use the vaginal contraceptive ring	0	0		0	0	
I do not like needles or injections	29 (47.5%)	23 (56.1%)	0.4	36 (46.8%)	16 (64%)	0.1

*p* values obtained from chi-squared test, Fisher’s exact test, and Student’s *t* test

^*^Single-item post-traumatic stress disorder screener

**Table 3:** Occupational determinants

	Intention to use PrEP			Condom use		
	Intenders (*n* = 61)	Non-intenders (*n* = 41)	*p* value	Consistent (*n* = 77)	Inconsistent (*n* = 25)	*p* value
Sex work characteristics in the previous three months						
Street-based work	41 (67.2%)	32 (78%)	0.2	48 (64%)	24 (96%)	**0.002**
Average number of clients per day (mean ± SD)	6.2 ± 4.3	5.9 ± 4.5	0.7	5.4 ± 3.8	8.2 ± 5.2	**0.004**
Average monthly income (mean ± SD)	125.2 ± 87.2	100.9 ± 66.1	0.1	115.3 ± 83.5	114.8 ± 69.7	0.9
Relationship with clients in the last 3 months						
Condomless sex	18(29.5%)	7(17.1%)	0.2			
Drug use with clients before or during sex	29 (47.5%)	17 (41.4%)	0.5	27 (35%)	19 (76%)	**< 0.001**
Condomless sex associated to drug use with clients	6/23 (26%)	1/13 (7.7%)	0.18			
Condom rupture	20/59 (33.9%)	7/40 (17.1%)	**0.07**	18/75 (24%)	9/24 (36%)	0.2
Condom coercion	34 (55.7%)	19 (46.3%)	0.35	34 (44.2%)	19 (76%)	**0.006**
Violence (verbal or physical) exerted clients	31 (50.8%)	22 (53.6%)	0.8	36 (46.5%)	17 (68%)	**0.06**
Clients with drug injection habits	11 (18%)	4 (9.8%)	0.2	4 (5.2%)	11(44%)	**< 0.001**

*p* values obtained from chi-squared test, Fisher’s exact test, and Student’s *t* test

Bold values indicate statistical significance (*p* value < 0.05)

**Table 4:** Social determinants

	Intention to use PrEP			Condom use		
	Intenders (*n* = 61)	Non-intenders (*n* = 41)	*p* value	Consistent (*n* = 77)	Inconsistent (*n* = 25)	*p* value
Relationships with stable sexual partner(s) in the last 3 months						
Stable sexual partner	28 (45.9%)	16 (39%)	0.5	33 (42.8%)	11 (44%)	0.9
Open relationship	3 (4.9%)	2 (4.9%)	1	3 (3.9%)	2 (8%)	0.5
Condomless sex	22 (34.4%)	13 (31.7%)	0.6	52 (67.5%)	14 (56%)	0.2
Violence (verbal or physical) exerted by partner	8 (13.1%)	4 (9.8%)	0.6	5 (6.5%)	7 (28%)	**0.004**
Dependence on external influence						
My partner would not allow me to take PrEP	8/28 (28.6%)	4/16 (25%)	0.7	11 (14.3%)	1 (4%)	0.2
I worry about being forced to condomless sex if I take PrEP	12 (19.7%)	8 (19.5%)	0.9	13 (16.7%)	7 (28%)	0.2
HIV-related stigma						
People will think I have HIV if I take PrEP	33 (54.1%)	24 (58.5%)	0.6	41 (53.3%)	16 (64%)	0.3
PrEP-related stigma						
Work colleagues will think less of me if I take PrEP	29/60 (48.3%)	14 (34.1%)	0.2	34 (44.1%)	9 (36%)	0.6
I would give a good example if I took medication to prevent HIV infection	52/59 (88.1%)	29/39 (74.4%)	0.08	58 (75.3%)	23 (92%)	0.2
I would tell others about using PrEP	47 (773%)	30/40 (75%)	0.8	56/76 (73.7%)	21 (84%)	0.3

*p* values obtained from chi-squared test, Fisher’s exact test, and Student’s *t* test

Bold value indicates statistical significance (*p* value < 0.05)

**Table 5:** Structural determinants

	Intention to use PrEP			Condom use		
	Intenders (*n* = 61)	Non-intenders (*n* = 41)	*p* value	Consistent (*n* = 77)	Inconsistent (*n* = 25)	*p* value
Migratory status						
Migrant	35 (57.4%)	31 (75.6%)	**0.05**	57 (74%)	9 (36%)	**0.001**
Legal status in Spain	49 (80.3%)	35 (85.4%)	0.8	60 (80%)	23 (92%)	0.1
Interaction with police in the last year						
Police detention	22 (36.1%)	14 (34.1%)	0.8	21 (27.2%)	15 (60%)	**0.003**
Change of workplace due to police presence*	15 (24.5%)	12 (29.2%)	0.6	19 (24.7%)	8 (32%)	0.4
Rushed negotiations with clients due to police presence*	16 (27.1%)	14 (31.4%)	0.8	17 (35.4%)	13/24 (54.2%)	0.1
Access to housing, health and social services						
Homelessness in the last 3 months	27 (44,3%)	19 (32.2%)	0.8	26 (33.8%)	20 (80%)	**< 0.001**
Attends primary healthcare center	26 (42.6%)	24 (58.5%)	0.1	43 (57.4%)	7 (28%)	**0.01**
Enrolled in harm reduction services	10 (16.4%)	6 (14.6%)	0.9	9 (11.7%)	7 (28%)	**0.05**
Receptor of community or NGO services	33 (54.1%)	23 (56.1%)	0.8	35 (45.5%)	21 (84%)	**0.001**
Barriers to healthcare						
Going to the healthcare center is too expensive	10/58 (17.2%)	2/39 (5.1%)	0.1	8 (10.3%)	4 (16%)	0.5
Healthcare personnel do not treat me well	9/59 (15.3%)	3 (7.3%)	0.2	10 (13%)	2 (8%)	0.7
I tell my physician I am a sex worker when it is appropriate	25 (40.7%)	21 (51.2%)	0.3	33 (42.8%)	13 (52%)	0.9
I would only go to the doctor if ill	34 (55.7%)	24 (58.5%)	0.9	42 (54.5%)	16 (64%)	0.4
My work schedule interferes with my ability to go to the healthcare center	5/60 (8.4%)	5/40 (12.5%)	0.5	7 (9%)	3 (12%)	0.7
I would not mind attending medical follow up if I took PrEP	57 (93.4%)	33/39 (84.6%)	0.2	69/75 (92%)	21 (84%)	0.2
I fear physicians and diagnostic tests	11 (18%)	9 (22%)	0.6	15 (19.4%)	5 (20%)	0.9

*p* values obtained from chi-squared test, Fisher’s exact test, and Student’s *t* test

^*^Only relevant for street-based sex workers

Bold values indicate statistical significance (*p* value < 0.05)

#### Correlation Matrix (Step 2)

In the correlation matrix for intention to use oral PrEP, there was a high correlation between (1) I would feel protected against HIV by taking PrEP, and (2) I would take PrEP even if it did not protect 100% against HIV infection, for which only the first variable was kept. In addition, the variable (1) I do not worry about HIV infection, and therefore, I do not want to take PrEP, was highly correlated to the variable; (2) I always use condoms, and therefore, I do not need to use PrEP, and only the first variable was included in subsequent analysis.

#### Multivariate Analyses (Step 3)

**Fig. 2:**
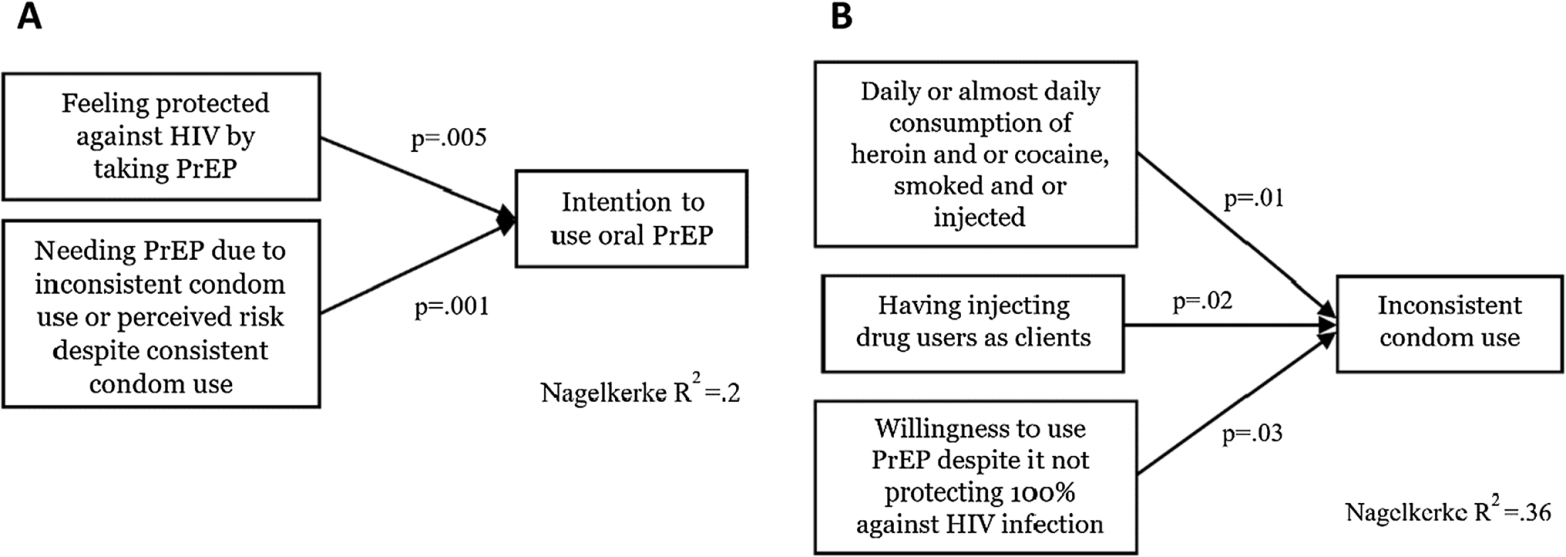
**A**, **B**. Final model for intention to use oral PrEP and condom use. The independent variables included were significantly associated in the two, backward and forward, regression models

In the stepwise regression models, feeling protected against HIV by taking PrEP, and participants’ perception that condom use did not offer sufficient protection against HIV infection were significantly associated with higher intention to use oral PrEP (Nagelkerke R2 = 0.20) (see Fig. 2A). Willingness to take PrEP even if it does not protect 100% against HIV infection was also included as an independent variable in the models but was not significantly associated with intention to use PrEP in either of the two, backward and forward, regression models.

### PrEP Delivery Preferences

For each participant, a preferred delivery method was determined based on the comparison of the Likert scale answers for each option, selecting that for which the participant had reported the highest level of interest. The preferred PrEP delivery method was injection (63.0%), followed by oral pills (20.6%), and vaginal ring (9.8%). The preference for injected PrEP was not associated with the workplace or difficulty taking pills daily. Women who expressed intention to use vaginal PrEP were more likely to be willing to take PrEP even if the protection level against HIV infection was below 100% (91.3% vs. 71.8%). More than half of participants (65.7%) preferred for PrEP to be delivered to them by a mobile unit on a monthly basis (60.8%), and half of them would take it indefinitely (52.0%) “while working in sex trade.”

### Determinants of Condom Use

#### Bivariate Analysis (Step 1)

Inconsistent condom use was significantly more common among street-based FSWs (32.1% vs. 3.4%). With regard to individual determinants, women who reported inconsistent condom use were more likely than women who reported consistent condom use to use cocaine (84.0% vs. 35.1%) and heroin (65.0% vs. 22.1%) on a daily or almost daily basis (91.7% vs. 32.2%), by smoking (88.0% vs. 29.8%) or injecting it (24.0% vs. 6.5%). They were also more likely to suffer from moderate to severe depression (60.0% vs. 27.3%), to report self-perceived HIV risk (52.0% vs. 24.6%), and to report at least one STI diagnosed in the previous year (52.0% vs. 14.3%). The participants who reported inconsistent condom use were more likely to lack a place to keep the pills (44.0% vs. 14.3%) and to express interest in PrEP even if it offered protection below 100% against HIV (92.0% vs. 70.2%) (Table 2).

With regard to occupational and social determinants, women who reported inconsistent condom use were more likely to practice sex work in the street (96% vs. 64%), have a higher average of daily clients (8.2 ± 5.2 vs. 5.4 ± 3.8), use drugs and/or alcohol with clients before or during sex (76.0% vs 35.0%), experience condom coercion by clients (76.0% vs. 44.2%), and engage with clients who use drugs by injection (44.0% vs. 5.2%). They were also more likely to report physical or verbal violence from stable sexual partners (28.0% vs. 6.5%) (Tables 3 and 4). Finally, with regard to structural determinants, women who reported inconsistent condom use were more likely to report homelessness (80.0% vs. 33.8%), to have been arrested by the police (60.0% vs. 27.3%), and to be enrolled in harm reduction services (28.0% vs. 11.7%). They were less likely to use primary care services (28.0% vs. 57.4%) (Table 5).

#### Correlation Matrix (Step 2)

In the correlation matrix for condom use, there was a high correlation between: (1) use of recreational drugs, (2) use of cocaine, (3) use of heroin, (4) use of smoked drugs, and (5) daily or almost daily use of drugs, for which only the last variable was kept. Other collinearities detected in the correlation matrix for intention to use oral PrEP and condom use were not acted upon as the variables included portrayed different information and did not interfere with the model.

#### Multivariate Analysis (Step 3)

In the stepwise regression models, daily or almost daily consumption of drugs, sexual encounters with clients who inject drugs, and willingness to take PrEP even if it does not protect 100% against HIV infection were significantly associated with a higher risk of inconsistent condom use (Nagelkerke R2 = 0.43) (see Fig. 2B). The number of daily clients was included as the independent variable in the models but was not significantly associated with intention to use PrEP in neither of the two regression models.

## Discussion

This study set out to identify the determinants of intention to use PrEP among a sample FSWs in Madrid, Spain, reached through field activities by local NGOs, in parallel with the determinants of condom use, the risk factor that qualifies FSWs for subsidized PrEP in Spain. To our knowledge, this is the first study to describe individual, occupational, social, and structural barriers and facilitators of intention to use PrEP among FSWs in Spain and the rest of Europe. Importantly, the study sample overrepresented street-based FSWs, which made up three-quarters of the participants. We found that PrEP awareness was low among study participants, but, after a brief introduction, intention to use PrEP was high. Intention to use oral PrEP did not correlate with social and structural determinants but with participants’ feeling protected with condoms and PrEP. In addition, we identified that even though oral PrEP was well accepted by FSWs, the preferred method for PrEP delivery was by injection provided by mobile units. Finally, inconsistent condom use was associated with frequent consumption of heroin/cocaine, having clients who inject drugs, and willingness to take PrEP even if it does not always protect against HIV transmission.

### Intention to Use PrEP and PrEP Delivery Methods

FSWs’ intention to use oral PrEP in our study sample was associated with individual determinants that might be influenced through education on HIV prevention. Our results thus offer explicit targets for future interventional studies (i.e., fostering the feeling of protection from PrEP and consistent condom use). This finding aligns with a previous qualitative study conducted among FSWs in South Africa, in which printed informational, educational, and communication materials influenced the decision to initiate PrEP in a majority of participants (87.9%) (Pillay et al., 2020). Indeed, educational strategies have been an important part of PrEP programs for FSWs that have achieved some degree of success. The Ashodaya project in India, with high rates of PrEP uptake, adherence, and retention (> 90% at 12 months), attributed its success to having a strong community program and carrying on group and individual counseling by peer educators that foster community norms around PrEP and condom use (Reza-Paul et al., 2020). An alternative successful program took place at Ministry of Health-run clinics in Senegal (82.4% uptake, 73.4% and retention at 12 months), using already established clinic programs for registered FSWs that include mandatory monthly check-ups and on-site education on STIs (Sarr et al., 2020). On the other hand, programs with a lower impact, such as the CHAMP Program in Cameroon (45% uptake, and 5% retention at 12 months), are looking to work closely with community groups to improve awareness and knowledge of the benefits of PrEP to normalize its use as an effective means of HIV prevention (Ndenkeh et al., 2022). Build-upon these past studies, our results suggest that increasing awareness about PrEP and its efficacy might be the most effective way to reinforce the intention to use PrEP in FSWs from our study population.

Additionally, based on the epidemiology of HIV infection in the Spanish context, reinforcing the protective role of condoms might reduce demand for PrEP among FSWs who use them correctly and consistently, and are at low risk of HIV infection (i.e., no injecting drug habits). In our study, more than half of the FSWs who reported consistent condom use did not consider this barrier enough protection against HIV infection and expressed intention to take PrEP. Nevertheless, it is important to contemplate that this finding is possibly related to the high rates of condom rupture and condom coercion experienced by study participants. In fact, FSWs’ intention to use oral PrEP as a complement to condoms to protect themselves against events that are beyond their control, such as “condom bursting,” has already been described in previous qualitative research studies in South Africa and Zimbabwe (Pillay et al., 2020; Reza-Paul et al., 2020; Sarr et al., 2020). In these instances, the appropriateness of PrEP versus PEP should be considered case by case and discussed individually with each woman. To facilitate this discussion, it is of the utmost importance to improve the awareness and knowledge of existing PEP services, which were unknown to most study participants.

Contrary to other studies (Ghayda et al., 2020; Makhakhe et al., 2022), stigma was not identified as a relevant barrier to the intention to use oral PrEP. It should be explored whether this finding can be explained by the influence of structural or social factors and values specific to the Spanish context (i.e., sex work not being illegal, having universal access to health care). Nevertheless, the above finding only relates to intention to use PrEP. It is known that FSWs in Spain do suffer from stigma that can impact their access to PrEP services (Asociación Pro Derechos Humanos de Andalucía n.d., 2019) with more than half of the study participants not reporting their occupation to healthcare providers when it would be appropriate to do so. Some additional things to consider in future studies are the impact of side effects (not significant in the analysis, but found to be a primary reason to decline initiation or continuation of oral PrEP among FSWs in previous studies (Amogne et al., 2022; Ndenkeh et al., 2022; Reza-Paul et al., 2020; Sarr et al., 2020)), and the need for integration of family planning services within PrEP programs given the low pre-existing use of contraceptive methods other than condoms in the study sample.

In this study sample of FSWs working mostly outdoors who are at the intersection of multiple vulnerability factors, the determinants of intention to use PrEP were better explained by the Information-Motivation-Behavioral Skills model for PrEP uptake than the structural vulnerability theory. Nevertheless, the participants recognized structural issues that may impact their PrEP adherence, such as PrEP delivery method and place. While both oral PrEP and injected PrEP were well accepted by the study participants, the preferred method was injectable PrEP (still not available in Spain). A previous qualitative study among FSWs in the Dominican Republic and Tanzania also found a preference for injectable PrEP over oral PrEP, as it was expected to cause less stigma, be easier to adhere to, and have fewer side effects than daily pills (Mantsios et al., 2022). Contrary to what has been observed in other studies in diverse global settings (Shannon et al., 2018), there was low acceptance of vaginal PrEP. During the brief introduction to PrEP, participants were told that the risk reduction offered by the vaginal ring was inferior to that offered by oral and injectable PrEP, which probably impacted their preference. Moreover, participants’ perceptions could have differed if they were more familiar with contraceptive vaginal rings. Finally, participants expressed a preference for PrEP delivery by mobile units, which have been proven successful for the delivery of other HIV-preventive services in the Marconi Polygon and La Cañada Real (Valencia et al., 2018, 2022). This finding aligns higher PrEP uptake by FSWs in South Africa at mobile clinics compared to fixed clinics (Pillay et al., 2020). FSWs in this setting report that their preference for mobile clinics is based on an appreciation for peer approach and tailored services that promote trust and reduce stigma, compared to fixed clinics (Makhakhe et al., 2019).

### Condom Use

Inconsistent condom use occurred almost exclusively among street-based FSWs. With regard to the determinants of condom use, we identified that the FSWs from the study sample with daily or almost daily use of heroin and/or cocaine and those engaging in sexual encounters with people who inject drugs, tend to practice condomless sex with higher frequency than those that do not. These findings are consistent with previous studies highlighting the association between drug use and risky sexual behaviors (Heiligenberg et al., 2012). Second, FSWs using drugs need to maintain a high income that covers their costly drug addiction, and engaging in condomless sex might prevent them from losing clients (Selvey et al., 2018).

While injecting drug use is known to be the most important HIV risk factor among FSWs in Europe (Platt et al., 2013), our study highlights the risk of HIV infection through condomless sex among FSWs with dependence on heroin and/or cocaine, regardless of whether it is smoked or injected. In addition to the use of drugs, the bivariate analysis highlights the multiple layers of individual, occupational, social, and structural determinants that affected the study participants with inconsistent condom use and that are known to increase vulnerability to HIV infection among FSWs (Tables 1–4), such as having moderate to severe depression, being homeless, working in the street, not being connected to primary care, engaging in drug use before or during sexual encounters with clients, having a high number of clients per day, and being victims of condom coercion and violence (Shannon et al., 2018). These findings support the importance of building PrEP programs able to reach the most vulnerable women.

Contrary to the situation in other countries (Tomko et al., 2019), police enforcement did not appear to impact condom use, and, therefore, its associated HIV risk, in the study sample. This is in accordance with the fact that selling sex per se is not illegal in Spain. Previous European evidence has shown that countries that criminalize sex work have more sex workers living with HIV, possibly due to deteriorating working environments and scarce of prevention services such as HIV and STI periodic screening (Reeves et al., 2017).

### Limitations

The results of this study should be cautiously interpreted based on its limitations. First, the small sample size and convenience sampling make the extrapolation of the study results to the broader population of FSWs in Madrid difficult. Most importantly, the study sample overrepresents street-based FSWs, which reached 71.6% in the current study compared to a prevalence of 7.4% in a study from 2021 carried out in five Spanish communities, including Madrid (Olmo et al., 2021). HIV-preventive behaviors may differ significantly between indoor and outdoor FSWs. The positive counterpart is that this study allows a closer examination of a very vulnerable group of FSWs in Madrid. Secondly, the lack of validated surveys for HIV risk and barriers to PrEP awareness and adherence specifically developed for cisgender FSWs should also be considered when interpreting the results. For each determinant, we adapted the shortest available scale with the goal of including a diverse and informative set of variables at the expense of losing some precision which might also explain, in part, the relatively low percentage of variance explained for intention to use PrEP. Finally, intention to use PrEP does not always correlate with PrEP initiation and adherence (i.e., the so-called intention-behavior gap), meaning that determinants identified in our study might differ from the ones associated with effective PrEP usage (Ndenkeh et al., 2022; Sheeran & Webb, 2016; Witte et al., 2022).

### Conclusion

After the successful roll-out of PrEP among MSM in Spain in 2019, now is the time to expand PrEP services to other at-need populations. First, in this study sample of cisgender FSWs working mostly outdoors we found little evidence of PrEP awareness. Second, the perceived feeling of HIV protection obtained from condom use and/or PrEP use was individual determinants of intention to use oral PrEP among study participants, which can be leveraged through appropriate educational interventions. Third, in our study FSWs with frequent consumption of heroin and/or cocaine, either smoked or injected, were at the highest risk of HIV infection through condomless sex and should be prioritized by PrEP campaigns. Finally, to overcome the barriers specific to this study sample, mobile units working on HIV prevention would benefit from being provided with PrEP delivery services, and the incorporation of injectable PrEP to the national health system should be considered.

## Data Availability

Available at OSF open data repository (https://osf.io/94et3/?view_only=ab29200774d24a5ba659df8ae91d97a3).

## Code Availability

Available at OSF open data repository (https://osf.io/94et3/?view_only=ab29200774d24a5ba659df8ae91d97a3).
